# Research on the synergetic development of producer services and manufacturing in northwest China’s inland central cities—A case study of Lanzhou

**DOI:** 10.1371/journal.pone.0293637

**Published:** 2023-12-14

**Authors:** Weimin Gong, Zhibin Zhang, Ming Zhang, Jian Wang, Dan Men

**Affiliations:** 1 School of Tourism, Shandong Women’s University, Jinan, 250300, China; 2 College of Geography and Environmental Science, Northwest Normal University, Lanzhou, 730070, China; Qufu Normal University, CHINA

## Abstract

Under the post Ford flexible production organization system, the existence of specialized division of labor has made the forward and backward connections between the producer services as an intermediate input and the manufacturing increasingly close. With the proposal of the "Belt and Road" and the deepening of “The development of the western region in China”, the northwest region plays an increasingly important role in China’s regional development strategy, and the economic transformation, especially the transformation and upgrading of the manufacturing, and it is crucial to its completion of the strategic mission entrusted by the state. Taking Lanzhou City as a case area, based on the measurement of the cooperative development level of producer services and manufacturing, this paper explores the internal law of the synergetic development of producer services and manufacturing in the northwest inland central city. The study found: The overall synergy development level of producer services and manufacturing in Lanzhou has been significantly improved in the dynamic evolution process, and two industries are in the transition stage from the primary coordinated development to the intermediate coordinated development at present. The spatial layout of producer services and manufacturing in Lanzhou City has certain spatial proximity, and the overall presents a "center-periphery" spatial pattern with the main urban area as the main component. From the perspective of the synergistic development of producer services and manufacturing in Lanzhou, the two industries have initially formed an interactive relationship of two-way promotion at present, but there is a significant asymmetry in the two-way promoting effect of the two industries.

## Introduction

Driven by economic globalization and information technology development, the world economy has shown the characteristics of industrial flexibility and manufacturing service. In this process, the global industrial structure has gradually changed from the "industrial economy" dominated by resources and capital to the "service economy" dominated by technology and innovation [1]. In this context, the relationship between producer services and manufacturing has gradually changed from the initial dependence of producer services on manufacturing to the independent development of producer services, thus strengthening the "externalization" trend of production service supply [2]. In addition, because of the manufacturing has a strong industrial correlation, it cannot do without the support of producer services as intermediate inputs in the process of transformation and upgrading [3–5]. Therefore, the synergy development of producer services and manufacturing is a complex process [6,7], the two industry pay special attention to the synergistic effect between each other while enhancing their own development strength [8].

Ellison and Glaser were the first to put forward the view of industrial collaborative agglomeration and collaborative development, and found that diversified industries tend to form spatial co-agglomeration [9]. With the continuous advancement of global service trade liberalization and the development of global production network, the synergistic relationship between producer services and manufacturing has been paid more and more attention by domestic and foreign scholars. Based on industrial economics, new economic geography, industrial cluster theory and other disciplines and related theories, the existing research mainly discusses and analyzes the interactive relationship between producer services and manufacturing from the two levels of industry and space [10,11].

From the perspective of industry, based on the division of labor theory of classical economics and the transaction cost theory of new institutional economics, scholars have discussed the industrial correlation characteristics of producer services and manufacturing from the perspective of supply and demand. From the perspective of demand, as the "mother" of producer services, manufacturing is still the main object of its services, although the producer services is gradually independent in the process of industrial division of labor [12]. The increase in the demand for producer services by manufacturing will play a certain role in stimulating the development of producer services [13]. Cohen and Zysman pointed out that the important demand sector for the output of service industry is the manufacturing, especially the producer services, which has a strong dependence on the manufacturing [14]. By studying the relationship between the three industries, Klodt found that there is a strong correlation between the secondary industry and the tertiary industry, especially the producer services, which is an important component of the service industry, whose development momentum mainly comes from the manufacturing [15]. Lodefalk discussed and analyzed the correlation between manufacturing exports and service factor inputs and found that the strengthening of manufacturing export intensity strongly expanded the demand for services [16]. From the perspective of supply, scholars believe that producer services play a supporting and leading role in the transformation and upgrading of the manufacturing and the climbing of the value chain in the development process. By analyzing the role of producer services and other professional intermediate inputs in trade, Markusen found that producer services, with their specialized production services, accelerated the improvement of manufacturing production efficiency [17]. After quantitative transnational analysis of comprehensive productivity, Restuccia believes that producer services occupies a dominant position in the interactive relationship with the manufacturing, and the intermediate inputs it provides can effectively improve the production efficiency of the manufacturing [18]. In recent years, with the deepening of international division of labor and the formation of global value chain, the cooperation between producer services and manufacturing has gradually strengthened and shown a trend of industrial integration. By studying the relationship between manufacturing and producer services, Goldhar et al. found that the two industries showed a trend of mutual penetration in the development process [19]. Hemilae J analyzed the degree of integration between producer services and traditional manufacturing in China and OECD countries from the perspective of the integration of producer services and manufacturing, and believed that producer services not only developed themselves through coupling with manufacturing, but also promoted the improvement of manufacturing production efficiency and the climb of global value chain [20]. Wang Xiaobo found through his research that although there is a certain trend of integration between producer services and manufacturing in China, this integration is a low level integration with structural imbalance, and there are still differences in the integration degree between industries [21].

From the perspective of space, based on classical location theory and agglomeration economic theory, scholars have studied the spatial location characteristics of the interaction between producer services and manufacturing, and formed two completely different views. Among them, one view holds that producer service enterprises tend to be located in areas where manufacturing enterprises gather. Andersson et al. empirically verified the synergistic effect of spatial distribution between producer services and manufacturing by constructing a simultaneous equation model and taking Sweden as a case [22]. Ke S et al. analyzed the panel data of 286 cities in China from 2003 to 2008 by using variable estimation method, and found that the manufacturing exhibits characteristics of being adjacent to producer services [23]. Ji Yahui and Wang Haijiang et al. explored the spatial relationship between producer services and manufacturing in China by using spatial measurement methods, and the results showed that regional spatial combinations and synergistic agglomeration existed between them [24,25]. Based on urban cross-section data, Ji Xiangyu et al. analyzed the impact of the collaborative agglomeration of producer services and manufacturing on China’s urban innovation, and found that the collaborative agglomeration of the two significantly improved the level of urban innovation [26]. Sassen et al. believe that as the manufacturing enters the manufacturing complex characterized by "new industrial space", producer services are still concentrated in metropolitan areas, so the agglomeration of manufacturing cannot explain the regionality of services [27–29]. In terms of the process of industrial evolution, scholars’ different conclusions on the spatial correlation analysis of producer services and manufacturing are not opposites, but spatial reflections of the changes in the interactive relationship between producer services and manufacturing at different stages of economic development.

To sum up, with the development of knowledge-capital complex clusters and the evolution of industrial division of labor and specialization, existing research on the industrial synergy and spatial linkage between producer services and manufacturing enterprises within cities is still in the stage of active exploration. Due to the low availability of micro-scale industry data within cities, the traditional empirical studies on the synergetic development of producer services and manufacturing are mostly based on regional or urban scale, and relatively few studies have analyzed the process and mechanism of the synergetic development of producer services and manufacturing at the micro-scale inside cities. In addition, the existing research areas are mainly the whole of China or the developed coastal areas of eastern China. While the synergetic development of producer service industry and manufacturing industry in northwest inland central cities has certain similarities with the whole country or other regions, the two industries often have certain regional uniqueness in the process of synergetic development due to the influence of regional differences, industrial development status, historical roots and other factors. Therefore, this paper takes Lanzhou City, a central inland city in northwest China, as a case area to explore the synergetic developmen law of producer services and manufacturing at micro scale inside the city from two levels of industry and space. On the one hand, it enriches the research results of the synergetic development of producer services and manufacturing at micro scale inside cities; On the other hand, it has certain practical significance for the inland central cities of northwest China to build a new system of high-quality and efficient service industries and promote the coordinated development and transformation and upgrading of industries.

## Data sources and research methods

### Overview of the study area

**Fig 1:**
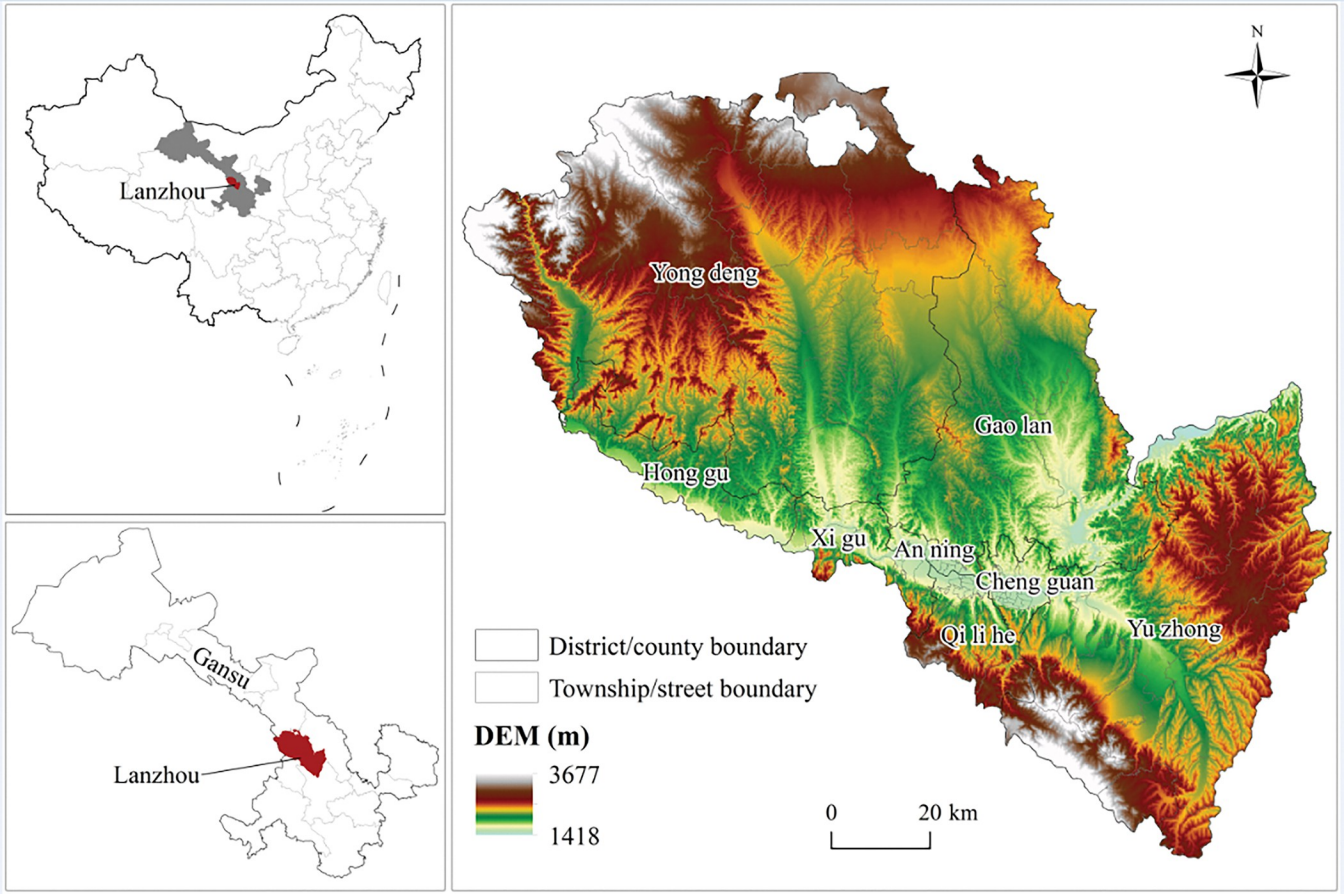
Lanzhou location map. Note: The basic map came from a public map from the standard map service website of the Ministry of Natural Resources of China. The drawing approval number is GS (2016)1593.

As the political and economic center of Gansu Province and the fortress of the ancient Silk Road, Lanzhou City has become an important transportation hub and commercial town in northwest China by virtue of its location advantage. It now has jurisdiction over 5 districts of Chengguan, Qilihe, Anning, Xigu and Honggu, and 3 counties of Yuzhong, Gaolan and Yongdeng (Fig 1). The Yellow River passes through the city from southwest to northeast, forming a beaded valley with alternating valleys and basins. The north and south mountains face each other in the main city of Lanzhou, forming a strip-shaped dumbbell valley basin about 35 kilometers long from east to west and 2–8 kilometers wide from north to south. With the proposal of the "Belt and Road" Initiative and the promotion of the new pattern of western development, as a comprehensive industrial city led by the petrochemical industry, which rose in the period of planned economy, Lanzhou is currently in a critical period of transition from mid-industrialization to late industrialization. The industrial structure of Lanzhou has gradually changed from production-oriented to service-oriented, and has now become a regional central city with important influence in the northwest inland [30]. According to the research needs and the regional reality of Lanzhou city, the main urban area located in the river valley is divided into the central area; Heping Town and Jinya Town in Yuzhong County, Zhonghe Town in Gaolan County, Wushui Town and Shuping Town in Yongdeng County, and Ping ’an Town in Honggu District are classified as suburban areas; The rest of the areas are the outer suburbs [31].

### Data sources

The basic data mainly comes from the registration data of industrial and commercial enterprises in Lanzhou from 1990 to 2020, including the attribute information of enterprise name, address, business scope, employment number, registration time and so on. After selecting and removing some enterprises with incomplete or incorrect information, the sample size of 139,168 enterprises was obtained, including 102,649 producer service enterprises and 7108 manufacturing enterprises. Through Baidu API interface, enterprise address information is transformed into enterprise spatial coordinates, so as to build a spatial database of Lanzhou producer services and manufacturing. In addition, the statistical data selected in this study are from the China Urban Statistical Yearbook (1990–2020), Gansu Development Yearbook (1990–2020), Lanzhou Statistical Yearbook (1990–2020), Lanzhou industrial and commercial enterprise registration data, and the first, second, third and fourth economic census data of Lanzhou. The administrative boundary data came from the national 1:100,000 basic geographic database of the National Basic Geographic Information Center.

### Research methods

#### The synergetic measurement model of grey related industries

Grey correlation analysis is a statistical method to measure the correlation degree of various factors according to the similarity degree of development between factors, focusing on the measurement of the dynamic change situation between systems. The degree of synergistic correlation between industries is considered by industry similarity, and the industries with higher similarity have stronger synergistic correlation. As a common method to measure the degree of correlation between elements, grey correlation analysis is calculated as follows [32]:

$$\gamma (j)(t)=\frac{{min}_{i}\;{min}_{j}|Z_{i}^{X}(t)-Z_{j}^{Y}(t)|+\rho {max}_{i}{max}_{j}|Z_{i}^{X}(t)-Z_{j}^{Y}(t)|}{\left|Z_{i}^{X}(t)-Z_{j}^{Y}(t)|+\rho {max}_{i}{max}_{j}|Z_{i}^{X}(t)-Z_{j}^{Y}(t)\right|}$$

Among them, *γ (j) (t)* represents the correlation coefficient at time *t*, and the larger the value, the stronger the inter-industry correlation. *Z*_*iX*_ *(t)* and *Z*_*iY*_ *(t)* respectively represent the standardized values of the indexes of industry *i* and industry *j* at time *t*, and *ρ* is the standardized coefficient.

**Nuclear density analysis.** Kernel density estimation method is often applied to the detection of spatial hot spots in order to analyze the spatial distribution characteristics of research objects. Based on reference to relevant literature [33], this paper adopts the kernel density estimation method to calculate the spatial distribution of producer services enterprises in Lanzhou at four time nodes. The formula is as follows:

$$\lambda_{h}=\sum_{i=1}^{n}\frac{3}{\pi h^{4}}{\left[1-\frac{{\left(s-s_{i}\right)}^{2}}{h^{2}}\right]}^{2}$$

Where *s* is the position of the enterprise and *s*_*i*_ is the position of the *i* th productive service enterprise within the radius of *s* as the center of the circle and *h* as the radius.

#### Analysis of regional agglomeration hotspots

The nearest distance hierarchical clustering method is usually used to identify clustering hotspots of different levels [34]. Its working principle is to define an aggregation unit, limit distance or threshold value and the minimum number of each aggregation unit according to the nearest distance of each enterprise point, and then compare the proximity distance between the aggregation unit and each point pair.

#### Action potency model

Considering the collinearity of variables and robustness of the model, this paper selects second-order least square method (*2SLS*) to measure and analyze the effect between producer service industry and manufacturing industry in Lanzhou [35]. The two-order regression equation is as follows:

First order regression::

$$\ln L_{T}=\alpha_{0}+\alpha_{1}\ln\hat{P_{p}}+\alpha_{2}\ln\hat{H}+\alpha_{3}\ln\hat{B}+\alpha \ln\hat{F}+\mu$$

Second order regression:

$$\ln P_{M}=\beta_{0}+\beta_{1}\ln L_{T}+\beta_{2}\ln P_{p}+\beta_{3}\ln H+\beta_{4}\ln B+\beta_{5}\ln F+\mu {+\beta }_{i}\nu$$

Where, *Pp* is the development level of producer service industry, *P*_*M*_ is the development level of manufacturing, freight volume *L*_*T*_ is the instrumental variable, and human capital *H*, infrastructure investment *B* and foreign direct investment *F* are the control variables. In order to objectively reflect the evolution of the function and effectiveness between producer services and manufacturing industry in Lanzhou in a long time series, this paper selects 1990, 2000, 2010 and 2020 as the measurement time nodes to conduct panel analysis of the function and effectiveness between producer services and manufacturing industry in the research period from 1990 to 2020.

## Measurement of the synergetic development of producer services and manufacturing in Lanzhou

In the composite system between producer services and manufacturing, there is a strong correlation between the two systems and each index element, and it shows obvious regularity [36]. In order to clarify the level of collaborative development and the process of collaborative evolution of producer services and manufacturing in Lanzhou, this paper measures and analyzes the industrial synergetic development of producer services and manufacturing from two aspects: the internal coordination of industry itself and the degree of synergy between industries.

### Construction of index system and classification of synergy types

#### Index system construction

In order to reflect the synergetic development process of producer services and manufacturing more comprehensively and reasonably, the synergetic development level evaluation index system of producer services and manufacturing is constructed from four aspects (Table 1): industrial scale, economic benefit, growth potential and social contribution [37].

**Table 1:** Indicators to measure the level of synergy between producer services and manufacturing.

Industry type	Primary index	Secondary index	Index interpretation
Producer services	Industrial scale	Number of enterprises	Total number of producer service enterprises
		Investment in fixed assets	Total fixed asset investment in producer services
		Industrial added value	Total value added of producer services industry
	Economic benefit	Average labor remuneration for employed persons	Overall wages in producer services/Number of employees in producer services
		Labor productivity	Total industrial value added of producer services/Number of employed people in producer services
	Growth potential	Value added growth rate	(Sales value of productive service industry in the current year/sales value of productive service industry in the previous year -1) * 100%
		Investment accounted for the proportion of total social investment	(fixed assets investment of producer services/fixed assets investment of the city) * 100%
	Social contribution	Employment number	Number of employees in the productive service industry
		Total profit and tax	Total tax revenue from productive services
Manufacturing	Industrial scale	Number of enterprises	Total number of manufacturing enterprises
		Investment in fixed assets	Total fixed asset investment in manufacturing industry
		Industrial sales value	Total sales value of manufacturing industry
	Economic benefit	Average labor remuneration for employed persons	Overall salary of manufacturing industry/number of manufacturing employees
		Labor productivity	Total value added of manufacturing industry/Number of employment in manufacturing industry
	Growth potential	Industrial sales output growth rate	(Manufacturing sales value of the year/manufacturing sales value of the previous year -1) *100%
		Proportion of investment in total social investment	(Manufacturing fixed asset investment/city fixed asset investment) *100% (Manufacturing fixed asset investment/city fixed asset investment
	Social contribution	Number of employed persons	Number of people employed in manufacturing
		Total profit and tax	Total manufacturing tax revenue

In the process of index selection, "industrial scale" is represented by the number of enterprise units, investment in fixed assets, sales output value of manufacturing or industrial added value of producer services, so as to reflect the total asset scale of producer services and manufacturing. "Industrial economic benefit" is represented by the average labor remuneration and labor productivity of employed people, in order to measure the respective profitability of producer services and manufacturing. When measuring the "growth potential" of industries, taking into account the attributes and characteristics of producer services and manufacturing industries, the "growth potential" of producer services is represented by the growth rate of industrial added value and the proportion of investment in the total social investment, while the "growth potential" of manufacturing is represented by the growth rate of industrial sales output value and the proportion of investment in the total social investment. The "social contribution" index of the industry is represented by the number of employees, the total profit and tax, and the profit and tax rate of the output value, so as to measure the contribution of the two industries in absorbing employment and increasing tax.

#### Classification of synergy types

First of all, based on the synergistic development value of producer services and manufacturing, the synergistic development degree of the two industries is roughly divided into three interval types: unacceptable interval (0~0.4), transitional interval (0.4~0.6) and acceptable interval (0.6~1). In order to have a more detailed grasp of the synergic development types of producer services and manufacturing, the unacceptable range is divided into four kinds of synergic types: extreme dysfunctional decline (0~0.1), severe dysfunctional decline (0.1~0.2), moderate dysfunctional decline (0.2 ~0.3) and mild dysfunctional decline (0.3~0.4). Secondly, considering that the high-quality synergic development of producer services and manufacturing is often based on the development of their own industries, the coordination degree of the industry itself should be measured and analyzed when measuring the degree of collaboration between the two industries. Based on this, according to the ratio of producer services self-coordination degree (*D*_*P*_) to manufacturing self-coordination degree (*D*_*M*_), the leading relationship between the two industries in the process of synergic development is further defined, and finally 30 composite types are divided [38].

#### Self-coordination analysis

**Fig 2:**
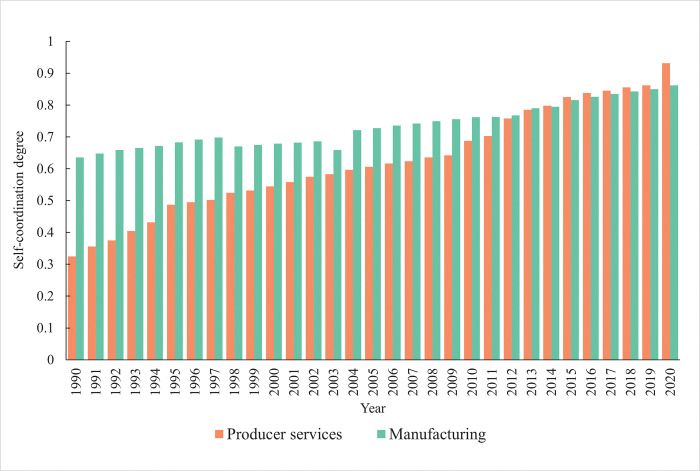
Coordination level between producer services and manufacturing in Lanzhou City.

The self-coordination level of producer services and manufacturing in Lanzhou has maintained a steady growth trend during the study period (Fig 2), indicating that the two industry have been in a dynamic evolution state in the process of their own industrial development. At the same time, with the evolution of industrial policy and industrial development stage, the self-coordination of the development of the two industries has also shown obvious stage characteristics. Through analysis, it is found that from the 1990s to the beginning of this century, Lanzhou’s manufacturing has always been leading the development of producer services with a high level of self coordination. The reason is that as an important national industrial base in northwest China, Lanzhou has experienced three stages of development, namely the construction of aid to the Soviet Union, the "The Third-Front Movement" (China’s strategic rear area construction centered on strengthening national defense in the 1960s to 1970s) and the reform and opening up, and has formed a good industrial foundation in the fields of general equipment manufacturing, electronic instrumentation, biomedics, and petrochemical industry. In addition, with its relatively complete industrial system, Lanzhou’s manufacturing has formed a strong industrial toughness, which has continuously enhanced its own development momentum and powerfully driven the development of producer services. Compared with the manufacturing, the producer services in Lanzhou in this period did not form a complete industrial chain and lacked independent innovation for the purpose of meeting the basic needs of the manufacturing, and its self-coordination level was low as a whole. In the analysis of the self-coordination level of producer services and manufacturing in Lanzhou, it is found that the growth trend of the coordination level of manufacturing in Lanzhou has gradually slowed down since 2000, while the coordination level of producer services has grown rapidly. Especially since 2014, the self-coordination level of producer services in Lanzhou experienced a rapid growth and has since maintained a leading position. During this process, the role of producer services as intermediate input in manufacturing development has been continuously refined. Simultaneously, the industrial system for producer services has been continuously enhanced and its self-coordination level has been effectively improved.

### Analysis of industrial synergy

**Fig 3:**
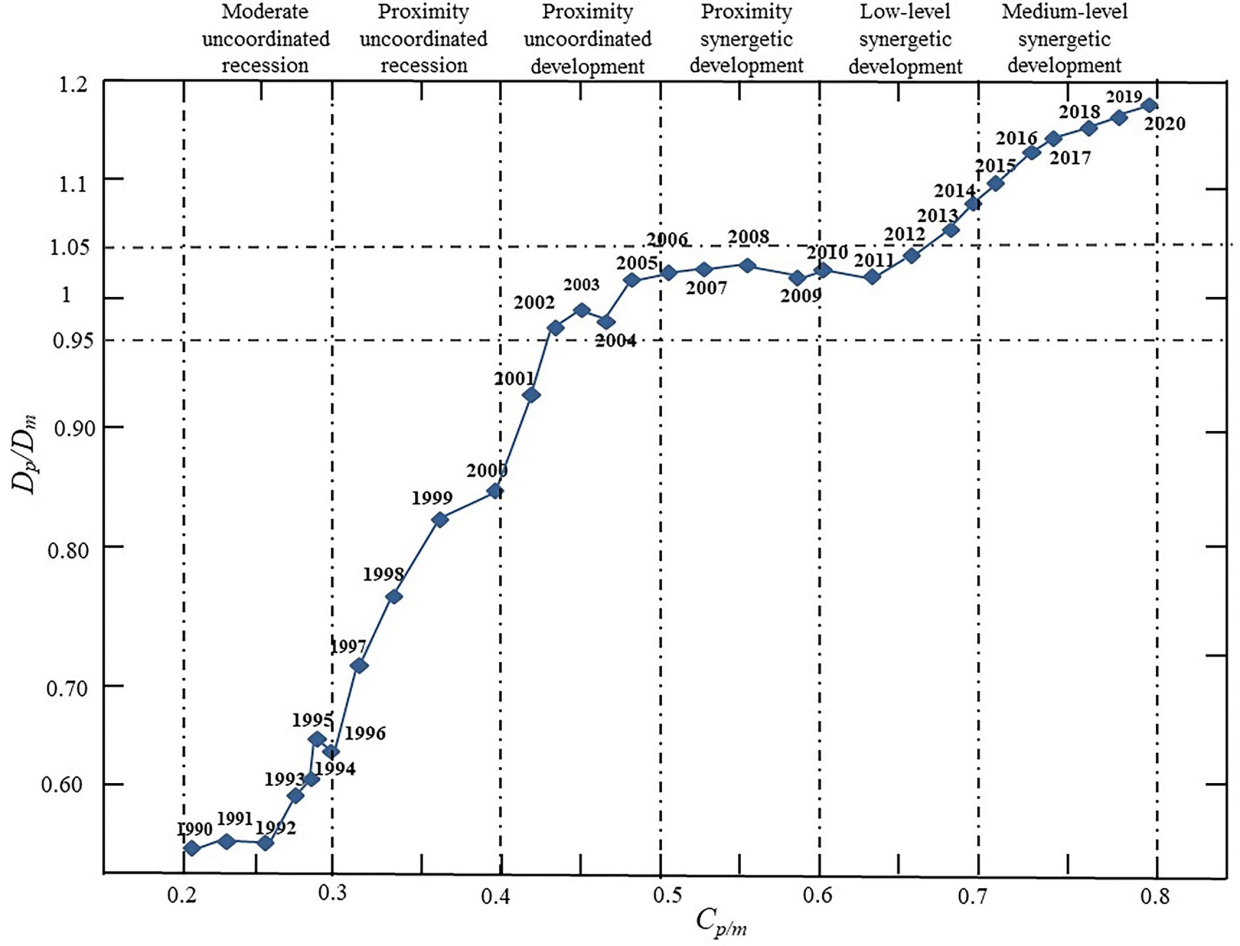
The coordinated development trend of producer services and manufacturing in Lanzhou. Note: *Dp* and *Dm* respectively represent the self-coordination level of producer services and manufacturing, *Cp/m* represents the synergetic development level of producer services and manufacturing.

The type of coordinated development of producer services and manufacturing in Lanzhou City changed three times in 2001, 2005 and 2010 (Fig 3). The measurement result indicate that the co-evolution process of producer services and manufacturing in Lanzhou can be categorized into the disordered recession period (1990–2005), the co-running-in period (2006–2010) and the synergetic development period (2011–2020).

During the disordered recession period from 1990 to 2005, the coupling coordination degree between producer services and manufacturing in Lanzhou fluctuated between 0.2 and 0.5, while the industrial coordination degree of both industries remaind low and experienced from moderate to mild disordered recession. Through further calculation, it is found that the level of coupling coordination between producer services and manufacturing in Lanzhou during this period is basically less than the collaboration threshold of 0.95, which indicates that the development of producer services lags behind that of manufacturing in this period. In the co-running-in period from 2006 to 2010, the Lanzhou producer services and manufacturing coupling coordination degree range from 0.4 to 0.6, and the ratio of their annual comprehensive development speed was in a steady growth state and gradually increased from the proximity uncoordinated development to the stage of Proximity synergetic development. By further measuring the ratio of annual comprehensive development speed of producer services and manufacturing in Lanzhou, it is found that the collaborative complex type of the two industries shift from a proximity uncoordinated development led by producer services to low-level synergetic development. During the synergetic development period from 2011 to 2020, the coupling coordination degree between producer services and manufacturing in Lanzhou is between 0.6 and 0.8, which is within the acceptable range of the synergetic development range. With the continuous development of producer services in Lanzhou and the continuous improvement of manufacturing ’s demand for producer services, the industrial coupling and coordination of the two industries have gradually developed from the low-level synergetic development to the stage of medium-level synergetic development. Especially since 2014, producer services in Lanzhou have played an increasingly prominent role in the development of manufacturing and gradually occupied a major position. Through measurement, it is found that in this period, the ratio of annual comprehensive development speed of producer services and manufacturing in Lanzhou was higher than the agglomeration threshold of 1.0, and the two type of development between them gradually evolved from the low-level synergetic development to the stage of medium-level synergetic developmen.

## Measurement of spatial collaborative development of producer services and manufacturing in Lanzhou

### Evolution of spatial pattern of producer services and manufacturing

**Fig 4:**
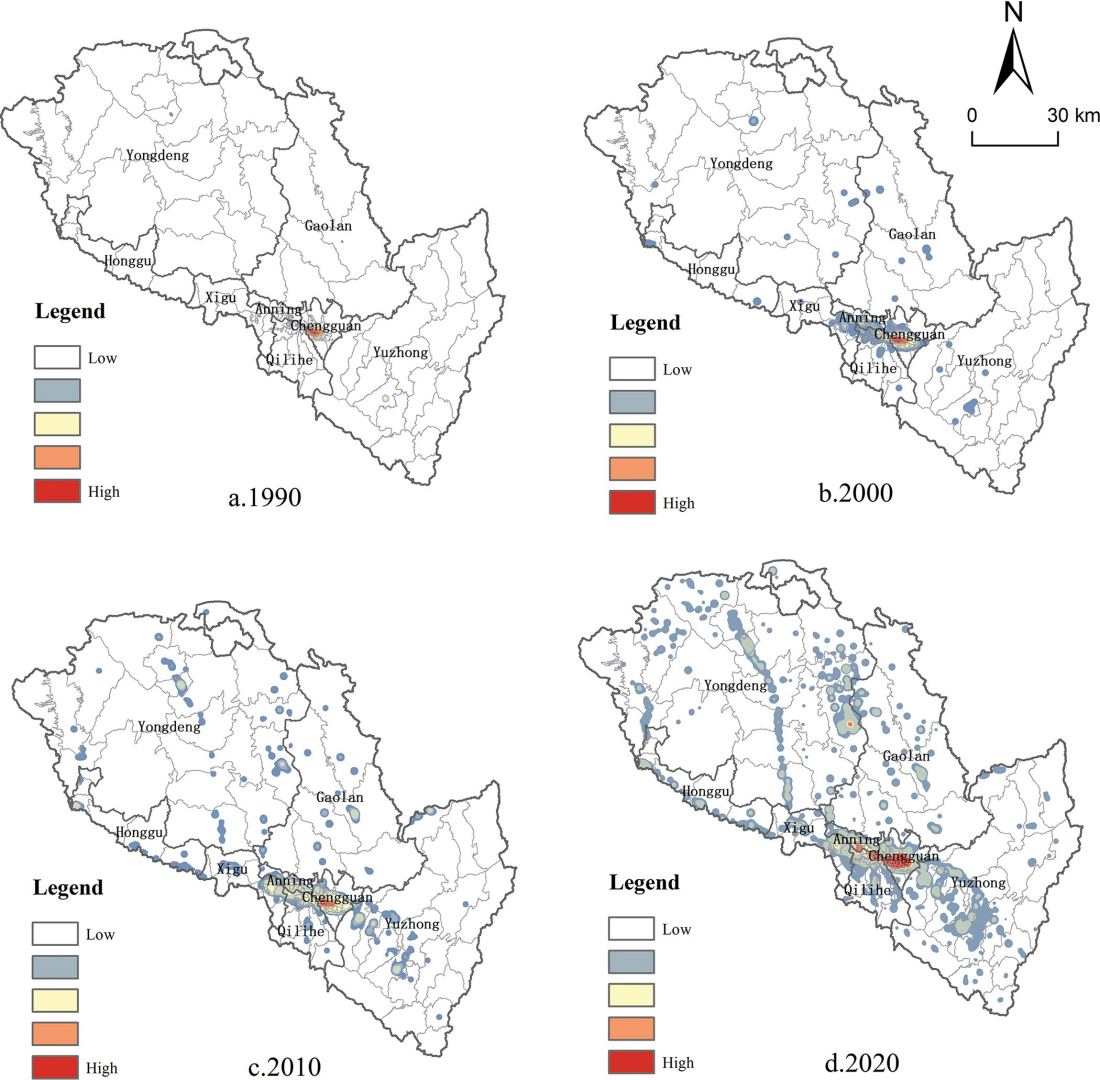
The spatial pattern of Lanzhou’s producer services from 1990 to 2020. Note: The basic map came from a public map from the standard map service website of the Ministry of Natural Resources of China. The drawing approval number is GS (2016)1593.

By conducting nuclear density analysis on the spatial pattern of producer services in Lanzhou, it is found that the producer services have evolved from an unbalanced single-center structure to a spatial pattern of "one heart, two wings and multiple groups". Before 1990, the development of producer services in Lanzhou was insufficient. Due to its spatial agglomeration characteristics, producer services were mainly distributed in the main urban area of the river valley,exhibiting the characteristics of single-center spatial agglomeration (Fig 4A). With the establishment of Lanzhou High-tech Development Zone, the development of numerous high-tech enterprises dominated by new materials, electronic information and software, and biotechnology has been strongly driven, which has injected new vitality into the development of producer services in Lanzhou. Following the year 2000, the implementation of the paid land use system resulted in the rental cost becoming a significant factor limiting the spatial arrangement of producer services. Consequently, the distribution density of producer services in the old city of Chengguan in Lanzhou experienced a decline. With the establishment of Lanzhou Economic and Technological Development Zone,which focuses on the development of industries such as electric machinery, new medicine and electronics, a new cluster of producer services has also been formed in Anning District. In addition, with the transformation and upgrading of the industrial structure, Qilihe District has gradually increased its demand for producer services based on its original manufacturing base, thus forming an industrial cluster of producer services related to the original manufacturing (Fig 4B). After 2010, with the development of Lanzhou New Area and the sub center of Yuzhong City, the original single center spatial pattern of Lanzhou’s producer services with the old city as the core was broken, and gradually developed into a multi center cluster pattern (Fig 4C). By 2020, the spatial pattern of producer services in Lanzhou has been further consolidated and basically formed a spatial pattern of "one heart, two wings and multiple groups". Among them, "One heart" refers to the main urban area located in the valley area, while "two wings" refer to Lanzhou New Area and Yuzhong Ecological Innovation City located in Qinwangchuan Basin and Yuzhong Basin respectively (Fig 4D).

**Fig 5:**
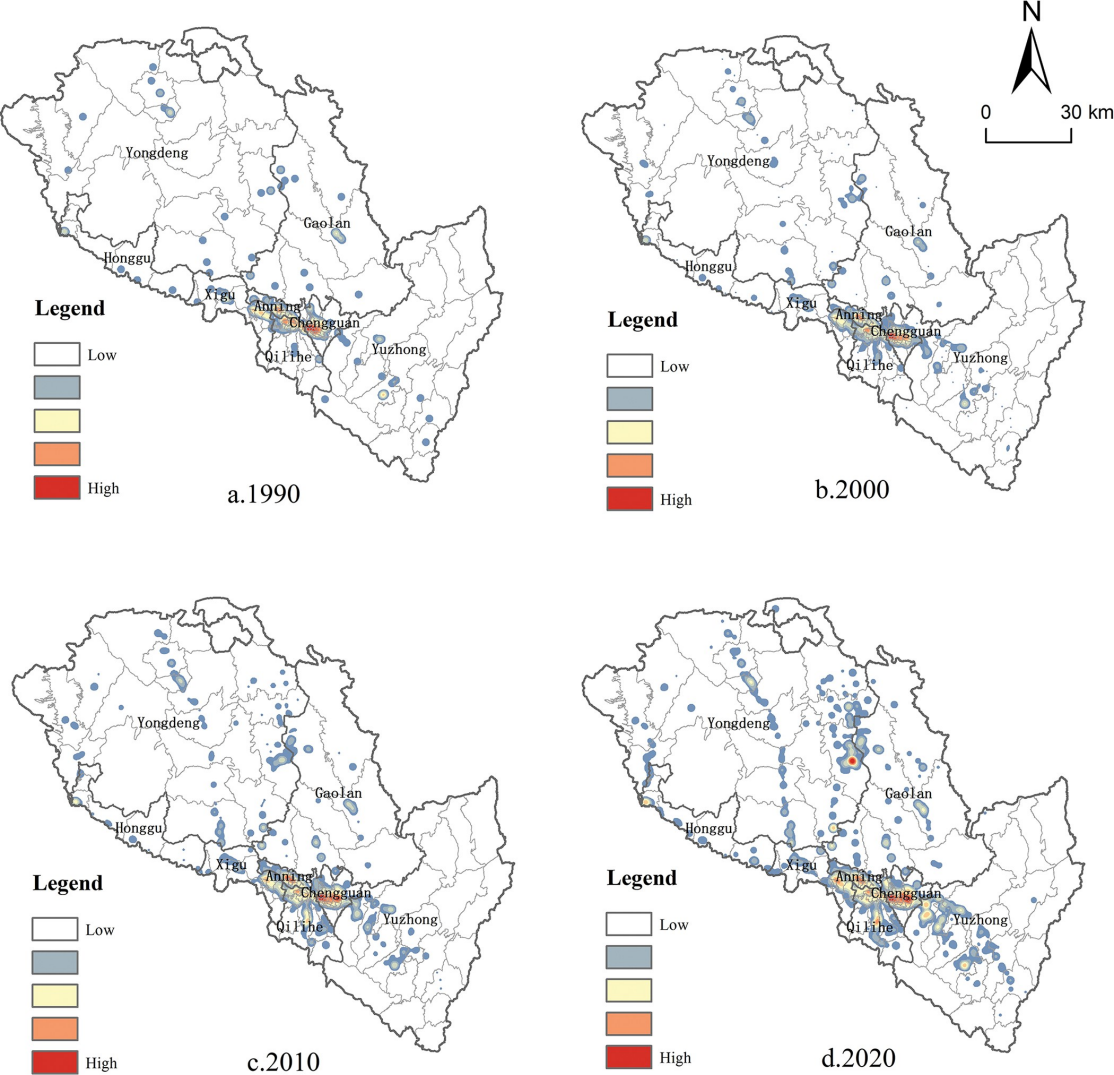
The spatial pattern of Lanzhou’s regional manufacturing from 1990 to 2020. Note: The basic map came from a public map from the standard map service website of the Ministry of Natural Resources of China. The drawing approval number is GS (2016)1593.

Nuclear density analysis reveals that the evolution of the spatial pattern of manufacturing in Lanzhou is gradually expanding from the main urban area to the urban periphery. Before 1990, the manufacturing in Lanzhou was mainly concentrated in the main urban areas of the river valley, such as the west of Chengguan District, the north of Qilihe District, the south of Anning District and the east of Xigu District. During this period, the manufacturing in Lanzhou established a singular center and polar core development pattern. (Fig 5A). Since 2000, manufacturing enterprises that were previously situated in urban areas have begun to relocate the suburbs, leading to the density of manufacturing enterprises located at the outer edge of the central urban area gradually increased (Fig 5B). In this process, the suburbs located on the periphery of the valley gradually evolved into the hot spots of stock transfer and incremental development of Lanzhou, and the manufacturing gradually concentrated in the suburbs under the promotion of the industrial development policy of "withdrawing from the secondary industry and developing the tertiary industry" in the urban area. Compared with the previous period, the spatial distribution pattern of the manufacturing in Lanzhou has not changed much in 2010, and the suburbanization trend of manufacturing location selection has been further reflected. With the establishment of Lanzhou New Area as a "state-level New area", the manufacturing has formed a cluster area in Lanzhou New Area located in the far suburbs, further promoting the development of the city’s industrial layout towards a multi-center model (Fig 5C). In 2020, the development and construction of Yuzhong Ecological Innovation City attracted manufacturing enterprises to settle in and formed a spatial pattern of "one main and two wings" with the main urban area and Lanzhou New Area (Fig 5D).

### Spatial agglomeration characteristics of producer services and manufacturing

**Fig 6:**
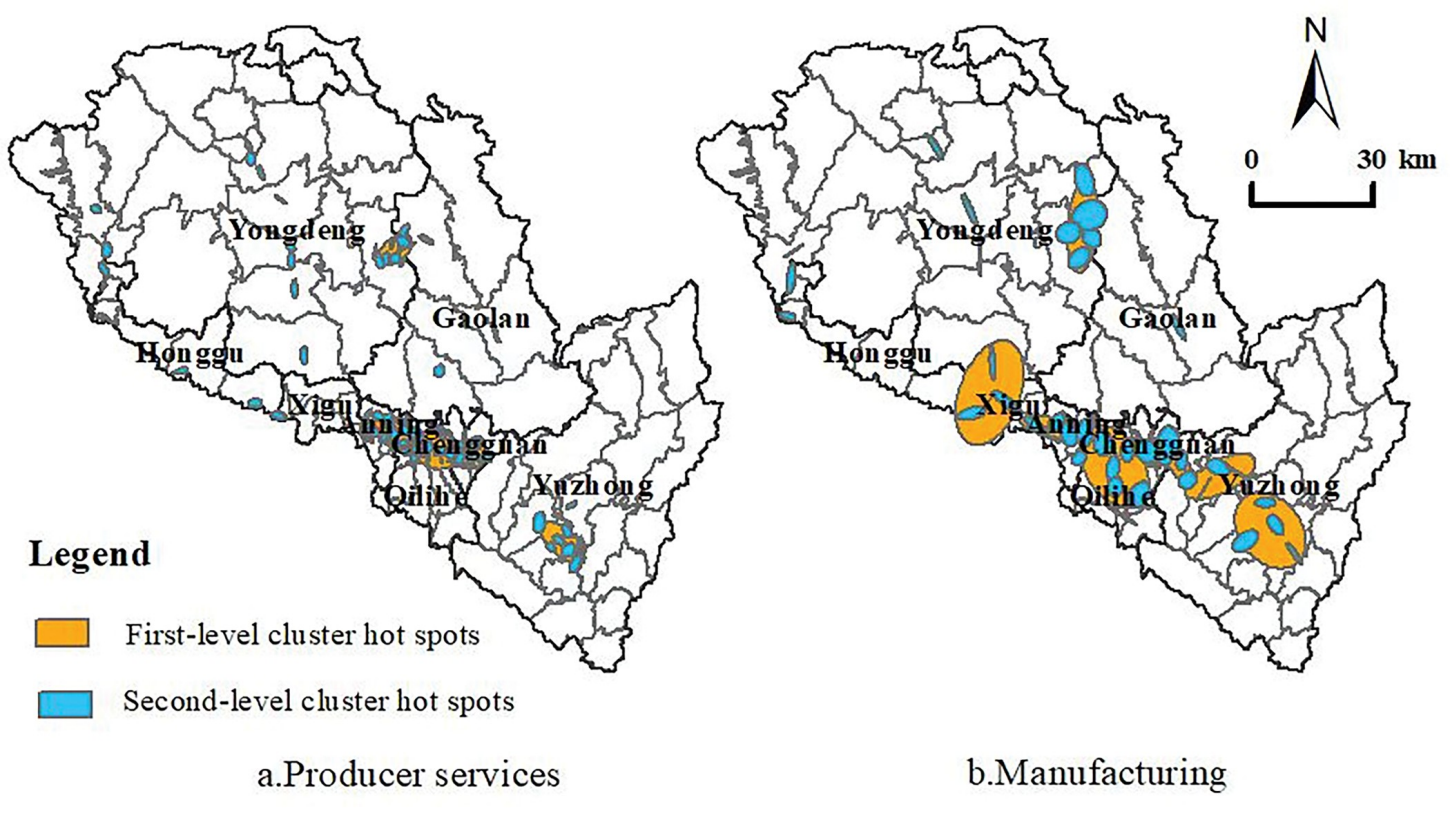
Spatial agglomeration and distribution of producer services and manufacturing in Lanzhou. Note: The basic map came from a public map from the standard map service website of the Ministry of Natural Resources of China. The drawing approval number is GS (2016)1593.

As a whole, the producer service enterprises in Lanzhou tend to be clustered and distributed in high density in the main urban area of the river valley. In addition, some large-scale producer services agglomeration areas have also formed in Lanzhou New Area, located in the outer suburbs of the city. With the acceleration of the construction process of Yuzhong Ecological Innovation City, a relatively dense producer service agglomeration area has been formed in Heping Town and Chengguan Town, which are located in the suburbs, showing the spatial distribution characteristics of "one heart, two wings and multiple groups". From the perspective of spatial distribution of agglomeration hotspots, the overall spatial agglomeration characteristics of producer service enterprises in Lanzhou are obvious, forming six primary agglomeration hotspots and 108 secondary agglomeration hotspots (Fig 6A). The agglomeration hotspots are distributed in the main urban area of the river valley and some streets of Lanzhou New District with a relatively high density, and the emerging agglomeration hotspots of producer service enterprises are formed in Heping Town and Chengguan Town, which are located in the suburbs. Among them, the first-level cluster hotspot area mainly includes the eastern cluster hotspot area with Heping as the core, the commercial center cluster hotspot area with Dongfanghong Square as the core, the emerging cluster hotspot area with Anning-Qilihe as the core, the old industrial zone with Xigu as the core, the new district cluster hotspot area with Rainbow City as the core and the new town cluster hotspot area with Chengguan town as the core. In addition, a number of secondary agglomeration hotspots have been formed in the periphery of the city with the Lianhuo Expressway as the development axis, which has become a new spatial carrier for the outward expansion of producer services.

Compared with the more concentrated distribution pattern of producer service enterprises, Lanzhou manufacturing enterprises have formed 7 first-level cluster hot spots and 37 second-level cluster hot spots, and the overall spatial distribution trend of suburbanization is obvious (Fig 6B). Lanzhou manufacturing enterprises are mainly distributed in the main city of the Xigu Old Industrial Zone and some towns located in the suburbs, such as Wushui town, Ping ’an Town, Xiguoyuan town, Heping town, etc. Among them, two of the first level agglomeration hotspots for manufacturing enterprises are located in the main urban area, and five are located in the suburbs; The distribution range of secondary hot spots in the suburbs is relatively wide, showing a spatial distribution pattern of diffusion from the main urban area to the outer circles of the city.

### Measurement of spatial correlation between producer services and manufacturing in Lanzhou City

Through the analysis of the evolution and agglomeration characteristics of the spatial pattern of producer services and manufacturing in Lanzhou city, it is found that the spatial pattern of the two has certain similarities as a whole, showing the "center-periphery" spatial characteristics with the main urban area as the main body and peripheral local areas as the supplement. In view of this, in order to further objectively reflect the geographical distribution of producer service industry and manufacturing industry in Lanzhou city, the district (township) is taken as the research unit to measure the geographical connection of the two industries in Lanzhou in 2020.

**Fig 7:**
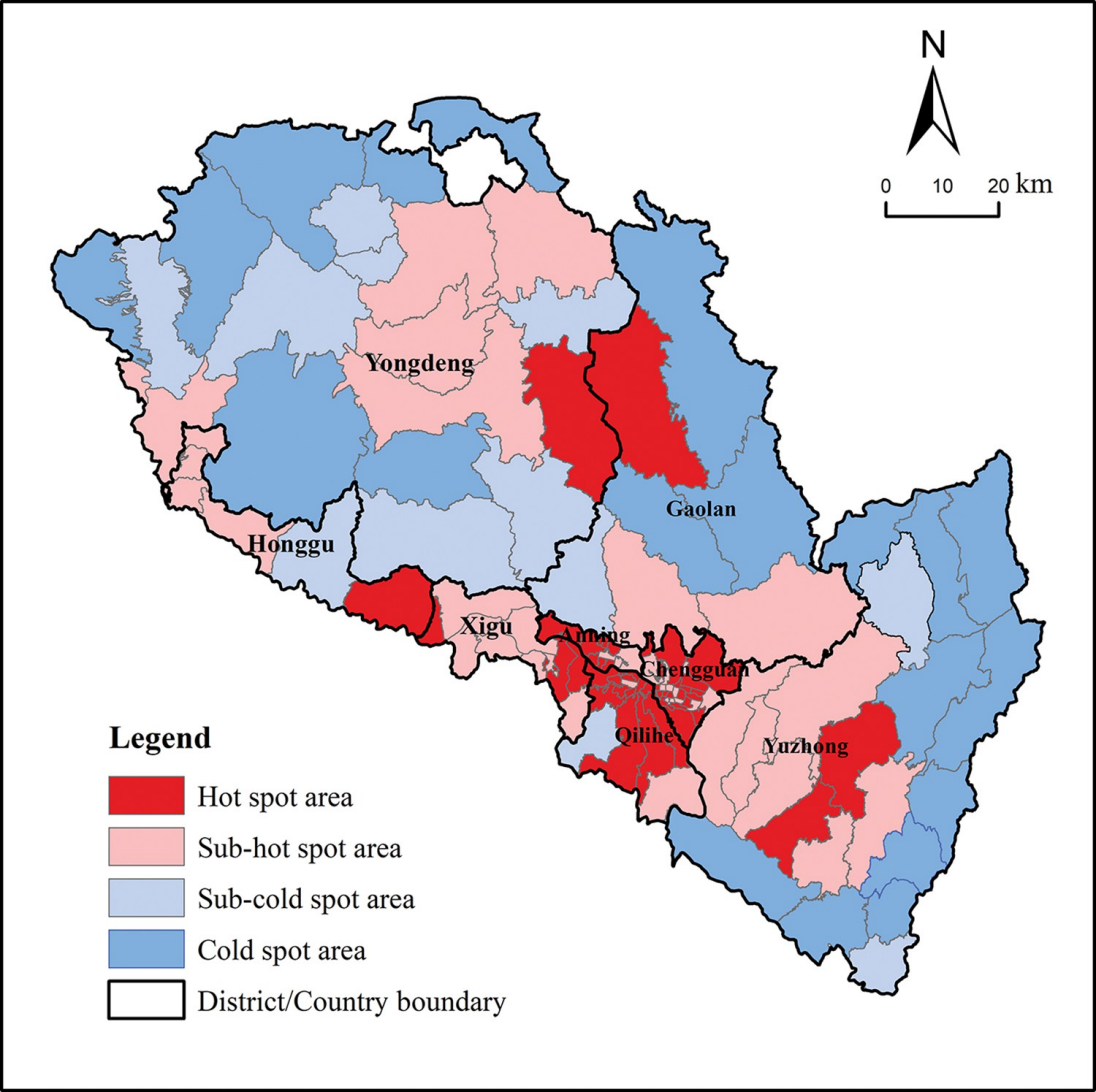
Hot spot map of geographical connection rate between producer services and manufacturing in Lanzhou. Note: The basic map came from a public map from the standard map service website of the Ministry of Natural Resources of China. The drawing approval number is GS (2016)1593.

Measurements reveal that the overall geographical linkage rate between producer services and manufacturing in Lanzhou is relatively high, reaching 78.6 in 2020. This indicates that the spatial interdependence of the two industries is relatively high due to joint action of supply and demand. The reason may be that the diversified services required by the agglomeration development of manufacturing industry will appeal to the local producer services. Meanwhile producer service enterprises tend to locate in the location with a high degree of agglomeration of manufacturing enterprises, leveraging the spatial spillover effect to serve the surrounding manufacturing and consequently achieving a larger market demand. From the spatial pattern of geographical linkage rate between producer services and manufacturing in Lanzhou, it is apparent there are obvious regional differences in their spatial correlation degree (Fig 7). The hot spots are mainly distributed in some streets of the main urban area, Lanzhou New Area, and Yuzhong Basin. The secondary hot spots are concentrated in Zhonghe Town and Shichuan of Gaolan County, Heping Town, Dingyuan Town, Jinya Town and Lianta Township of Yuzhong County, Haishiwan Town and Honggu Township of Honggu District, as well as Liushu Township and Datong Town of Gaolan County. The number of sub-cold spots is small and mainly concentrated in the suburbs of Gaolan County and the south of Yongdeng County. The cold spot area is mainly distributed in a few towns in Yongdeng County, Yuzhong County and Gaolan County, which are mostly located in the outer suburbs of the city. It can be seen that the central urban area of Lanzhou is still the main area for the synergistic agglomeration of manufacturing and producer services. However, due to the influence of the relocation of manufacturing, the synergistic agglomeration of producer services and manufacturing is not significant in some streets located in the river valley. Producer services and manufacturing in the outer areas of the main city synergistic agglomeration phenomenon is significant in Lanzhou. The reason may be that the suburbs are adjacent to the main urban area with relatively complete economic development factors and are distributed with a large number of manufacturing enterprises, thus attracting a large number of local manufacturing enterprises with industry-related producer service enterprises to enter. In the outer suburbs, the overall layout of manufacturing enterprises is relatively scattered, only in Lanzhou New Area and some areas of Yuzhong Ecological Innovation City to form a small range of agglomeration. This is mainly due to the limited attraction of producer service enterprises in the main urban area of Lanzhou, resulting in a low spatial correlation between producer services and manufacturing in this region.

## The synergistic development effect of producer services and manufacturing in Lanzhou

Based on the above analysis of industrial correlation and spatial proximity between producer services and manufacturing in Lanzhou, it is found that there is a significant correlation between the two industries in both industry and space. Under the bidirectional relationship between supply and demand, the producer services and manufacturing often have a bidirectional effect [35]. On the one hand, the development of producer services as an intermediate input industry has a certain promotion effect on the manufacturing; on the other hand, the development of manufacturing as the "mother" of producer services has a driving effect on producer services. In this paper, the second order least square method (*2SLS*) is used to analyze and measure the interaction between the producer services and manufacturing in Lanzhou.

### Indicator selection

With the refinement of industrial division of labor, although producer services has been gradually separated from manufacturing and developed into an independent industrial sector, it still has a close input-output relationship with manufacturing. Therefore, the realization of the interaction between producer services and manufacturing is the result of multiple factors working together. In view of this, in order to increase the accuracy of measurement results, this paper selects the development level of manufacturing (*P*_*M*_) as the explained variable to measure the promotion of manufacturing by producer services. The development level of producer services (*P*_*P*_) is selected as the main explanatory variable to measure the promotion of producer services to the development of manufacturing. Considering that the development of manufacturing is often affected by other factors besides producer services, in order to avoid the endogenous problem caused by the omission of indicators, the development level of logistics industry (*L*_*T*_) is selected as the instrumental variable in this paper. Among them, the development of manufacturing and the level of producer services are respectively characterized by the output value of the two industries. The development level of the logistics industry is characterized by freight volume. On this basis, in order to increase the robustness of the model, human capital (*H*), infrastructure investment (*B*), and foreign direct investment (*F*) are used as control variables. Among them, human capital is represented by the number of full-time teachers in colleges and universities, infrastructure investment is represented by the amount of regional fixed assets investment, and foreign direct investment is measured by the total amount of foreign direct investment.

### Regression results

#### The promotion effect of producer services to the development of manufacturing

As can be seen from the measurement results (Table 2), every 1% increase in the output value of producer services, the output value of manufacturing in the four time nodes increases by 0.535%, 0.582%, 0.602% and 0.635% respectively. This indicate that with the expansion of the development scale of producer services in Lanzhou, the development and technological innovation capability of manufacturing are effectively promoted. The impact of logistics industry output value on the manufacturing is gradually increasing, but the overall effectiveness still needs to be further improved. Among them, before 2000, the output value of the logistics industry did not pass the 10% significance test of α, indicating that its role in promoting the development of manufacturing is small. With the continuous improvement of the modern logistics system, the manufacturing in Lanzhou is increasingly dependent on the logistics industry in its development. Through measurement, it is found that every 1% increase in logistics output value of Lanzhou in 2010 and 2020 will increase manufacturing output value by 0.27% and 0.30% respectively. Although this variable only passes the significance test of 5% and 10% at the two time nodes, the measurement results show that the development of logistics industry can effectively reduce the production cost of manufacturing industry and promote the improvement of manufacturing output value. In addition, from the perspective of the three control variables, the impact of infrastructure investment on the output value of the manufacturing industry has gradually increased. At the four time nodes, every 1% increase in infrastructure investment in Lanzhou will increase the output value of the manufacturing industry by 0.24%, 0.28%, 0.35% and 0.42% respectively. The measurement results show that infrastructure investment provides a strong foundation for the development of Lanzhou’s manufacturing. The driving effect of foreign direct investment on the manufacturing in Lanzhou is not prominent. Through measurement, it is found that the effect coefficient of this factor on the manufacturing has gradually increased at the four time nodes, but it has not passed the significance test. This measurement result shows that as an inland city in northwest China, the manufacturing of Lanzhou is mainly dominated by local enterprises, which has a certain degree of enterprise embeddedness and isolation, and is not strongly dependent on foreign investment.

**Table 2:** Estimated results and robustness of manufacturing development promoted by producer. Services.

Variable	1990	2000	2010	2020
lnP_P_	0.535*	0.582*	0.602**	0.635***
	(3.25)	(2.95)	(5.20)	(4.39)
lnL_T_	0.282***	0.355**	0.362**	0.285**
	(3.02)	(2.39)	(4.12)	(5.10)
lnH	0.221	0.240	0.239	0.125
	(2.07)	(3.22)	(2.95)	(3.03)
lnB	0.240***	0.283***	0.355***	0.428***
	(5.06)	(4.52)	(3.85)	(5.44)
lnF	0.136	0.142	0.147*	0.158*
	(3.49)	(3.58)	(4.25)	(5.54)
R^2^	0.932	0.958	0.965	0.910
Hausman test	0.028	0.035	0.039	0.045

Note: The t statistic is in parentheses.

***, **, and * indicate that α is significant at the significance level of 1%, 5%, and 10%, respectively.

#### The driving effect of manufacturing on the development of producer services

The impact of Lanzhou’s manufacturing output value on the development of producer services is on the rise and has passed the significance test of 1% of the model since 2010 (Table 3). In 1990 and 2000, the impact of Lanzhou’s manufacturing output on producer services only passed the significance test of 10% of the model. In contrast, after 2010, the impact of the output value of Lanzhou’s manufacturing industry on producer services increased significantly and passed the significance test of 1% of the model. In contrast, since 2010, the impact of Lanzhou’s manufacturing output on producer services has increased significantly and passed the 1% significance test of the model.Specifically, for every 1% increase in the output value of Lanzhou’s manufacturing, the output value of producer services will increase by 0.702% and 0.735% respectively.This measurement result shows that the manufacturing in Lanzhou has a positive role in promoting producer services through the demand effect, and this role is gradually strengthened with the improvement of the development level and level of the manufacturing. On the whole, the manufacturing in Lanzhou has a strong driving effect on producer services, indicating that the manufacturing demand is dominant in the process of coordinated development of the two industries.

**Table 3:** Estimated results and robustness of manufacturing development to promote producer services.

Variable	1990	2000	2010	2020
LnP_M_	0.607* (5.085)	0.682* (8.995)	0.702*** (7.593)	0.735*** (6.658)
Lnh_M_	0.208 (1.59)	0.220 (2.59)	0.285* (1.08)	0.306** (1.47)
lnH	0.482* (3.285)	0.558** (4.054)	0.580*** (2.857)	0.621*** (5.262)
c	0.128 (0.204)	0.157 (0.355)	0.164 (0.273)	0.178 (0.198)
lnF	0.185* (4.857)	0.206* (3.905)	0.258* (2.414)	0.287** (3.282)
R^2^	0.970	0.988	0.915	0.980
Hausman test	0.028	0.035	0.039	0.045

Note: The t statistic is in parentheses.

***, **, and * indicate that α is significant at the significance level of 1%, 5%, and 10%, respectively.

The number of employees in manufacturing has a positive effect on the development of producer services, but its effect is different in different periods. Through measurement, it is found that in 1990 and 2000, the number of manufacturing employment in Lanzhou had a small impact on the output value of producer services and did not pass the model significance test. The reason may be that the low level of manufacturing development in Lanzhou during this period has less demand for producer services, and its effect needs to be further improved. Due to the advancement of industrialization and the growing need for producer services in manufacturing, the dependence of producer services in Lanzhou on manufacturing has increased significantly. The model measurement results show that the driving effect of Lanzhou’s manufacturing on producer services has passed the significance test of 5% and 10% in 2000 and 2020, respectively. Specifically, for every 1% increase in the number of manufacturing employment in Lanzhou, the output value of producer services increases by 0.285% and 0.306% respectively.

From the measurement results of the three control variables, human capital has effectively promoted the development of producer services in Lanzhou. Every 10% increase in human capital, the output value of producer services in Lanzhou increased by 0.482%, 0.558%, 0.580% and 0.621% respectively. In contrast, the impact of foreign direct investment on producer services in Lanzhou is not significant enough, and it only passed the model significance test of 10% before 2010. With the increasing efforts of the government to attract investment, although foreign capital has injected new impetus to stimulate the vitality of the urban economy, the role of foreign capital in Lanzhou in producer services in 2020 is not significant because of its small proportion in the total economic volume.

## Conclusion and prospect

### Research conclusion

Starting from the background of the new industrialization era characterized by the transformation from manufacturing to service-oriented manufacturing, this paper selects Lanzhou, the northwest central city of China along the "the Belt and Road", as the research object, and measures the degree of synergetic development of Lanzhou’s producer services and manufacturing from the perspective of industry and space. On this basis, the interactive relationship between the two industry was further verified. The main conclusions are as follows:

The overall synergetic development level of producer services and manufacturing in Lanzhou has been significantly improved in the dynamic evolution process. During the study period, the co-evolution process of producer services and manufacturing in Lanzhou City has experienced three stages: the disordered recession period (1990–2005), the co-running-in period (2006–2010) and the synergetic development period (2011–2020). At present, the producer services and manufacturing in Lanzhou are in the transition stage from primary to intermediate synergetic development.

The spatial layout of producer services and manufacturing in Lanzhou has a certain spatial proximity, and the overall presents a "center-periphery" spatial pattern supplemented by the main urban area. With the decentralization of urban service functions, producer services also presents a certain trend of suburbanization, but its scale and density in the suburbs are much lower than that of manufacturing.

Producer services and manufacturing in Lanzhou have formed an interactive relationship of mutual promotion. Producer services has an obvious promoting effect on the development of manufacturing,and manufacturing also has an obvious pulling effect on the development of producer services. At the same time, there is a significant asymmetry between the effects of producer services and manufacturing in Lanzhou, and the effect of producer services being pulled by manufacturing is more obvious.

### Research prospect

In the context of social transformation and the New normal of economy, China’s economic development has changed from focusing on quantity to focusing on quality. The "the Belt and Road Initiative" provides a rare historical opportunity for the economic development of the northwest inland central cities represented by Lanzhou. This paper takes Lanzhou as a case area to study the coordinated development of producer services and manufacturing in the northwest inland central city. Although some useful results have been achieved, the following aspects need further discussion.

With the deepening of China’s economic reform and the improvement of its political system, the government will further externalize various factor markets, and the government’s ability to allocate resources will also change. In addition, the central cities in the northwest inland region of China are influenced by factors such as history, location, and socio-economic factors, and have certain particularities. Therefore, in the face of the evolution of the relationship between the government and the market in the new era, how to make full use of the market mechanism and the government mechanism and build a high-quality industrial cooperative development system to realize the coordination and synergetic development among industries in the northwest inland central cities remains to be further discussed in the future research.

Although the producer services gradually becomes independent from the manufacturing in the process of industrial division of labor, the existence of industrial linkages makes it detour along the value chain and promotes the growth of added value in the manufacturing. It is precisely because of the existence of this correlation that the interaction between producer services and manufacturing has been deeply explored by many scholars. However, with the gradual refinement of the social division of labor, the service objects of producer services have also been greatly enriched, so that the meaning of producer services has gradually exceeded the basic meaning when it was separated from the manufacturing. Therefore, how to scientifically, reasonably and correctly grasp the relationship between the comprehensive concept and specific categories of producer services and its new meaning for manufacturing is an important direction in the future research.

In recent years, although the producer services has made significant progress in the northwest inland central cities of China represented by Lanzhou and gradually become an important force supporting its internal spatial structure during the transformation period, there is a significant gap in both industrial development level and industrial resilience compared to eastern coastal cities. Based on the regional resource factor endowment, how to further give play to the market mechanism while scientifically guiding the synergetic agglomeration of producer services enterprises and manufacturing enterprises in urban space, so as to improve the overall production efficiency of the city is also an important direction of future industrial spatial layout research. In view of this, before planning land use and investing in the construction of urban industrial functional zones, northwest inland cities of China must focus on guiding industrial clustering and intensive development, achieving coordinated development of urban functions, population distribution, resource environment, and industrial layout.

## Supporting information

S1 Data (RAR)
